# Efficacy and safety of disitamab vedotin in treatment of advanced gastric cancer based on real-world

**DOI:** 10.3389/fonc.2026.1807824

**Published:** 2026-05-20

**Authors:** Pan Cheng, Jianjuan Ge, Shuangjia Huang, Dongzan Yang, Shining Xie, Bowen Wang, Zhaoshi Bai, Jichen He, Xiaolin Liu

**Affiliations:** 1 Department of Pharmacy, Jingjiang People’s Hospital Affiliatedo Yangzhou University, Taizhou, China; 2 Department of Oncology, Affiliated Tumor Hospital of Nantong University, Nantong, China; 3 Jiangsu Cancer Hospital/The Affiliated Cancer Hospital of Nanjing Medical University/Jiangsu Institute of Cancer Research, Nanjing, China; 4 Department of Pharmacy, Puer People’s Hospital, Puer, China; 5 Department of Pharmacy, Beijing Ditan Hospital Affiliated to Capital Medical University Xuzhou Hospital (Xuzhou Seventh People’s Hospital), Xuzhou, China; 6 Department of Pharmacy, Zhoukou First People’s Hospital, Zhoukou, China

**Keywords:** disitamab vedotin, efficacy, gastric cancer, human epidermal growth factor 2, real-world research

## Abstract

**Objective:**

To evaluate the efficacy and safety of disitamab vedotin (RC48) in the treatment of advanced gastric cancer in real-world.

**Methods:**

A retrospective analysis was conducted on clinical data of patients with human epidermal growth factor receptor 2 (HER2)-expressing advanced gastric cancer who were treated with disitamab vedotin at our institution between October 2021 and October 2024. Clinical efficacy and adverse events were assessed. Factors influencing the efficacy of disitamab vedotin were evaluated using the Log-rank test for intergroup analysis.

**Results:**

A total of 58 patients with HER2-expressing advanced gastric cancer who received RC48 treatment were included in this study. The objective response rate (ORR) was 10.3%, and the disease control rate (DCR) was 63.8%. The median progression-free survival (mPFS) for all patients was 4.2 months (95% CI = 3.002-5.398), with a 1-year progression-free survival rate of 13.0%. Subgroup analysis revealed that DCR was significantly higher in patients receiving RC48 as third-line therapy compared to those receiving it as later-line therapy (75.0% *vs*. 50.0%, *P* = 0.049). The DCR was also significantly higher in patients who experienced adverse drug reactions (ADRs) than in those who did not (76.7% *vs*. 50.0%, *P* = 0.035). Male patients had a significantly longer mPFS than female patients (4.9 months *vs.* 2.6 months, Log-rank *P* = 0.006). Common RC48-related ADRs included neutropenia, hemoglobin reduction, and liver impairment. The incidence of grade III or higher ADRs was 17.2% (10/58). Univariate analysis indicated that the presence of underlying diseases and the line of RC48 therapy were significantly associated with the occurrence of ADRs.

**Conclusions:**

In the real-world setting, RC48 shows some clinical efficacy and a manageable safety profile in patients with HER2-expressing advanced gastric cancer. These findings provide useful information for clinical practice.

## 1 Introduction

Gastric cancer (GC) is one of the most common malignant tumors of the digestive tract worldwide. In 2022, China reported approximately 360,000 new cases of gastric cancer and 260,000 deaths, with its incidence and mortality rates ranking 5th and 3rd, respectively, among domestic malignant tumors (1). Human epidermal growth factor receptor 2 (HER2) is a member of the epidermal growth factor receptor family and is frequently expressed in various tumor tissues. It activates downstream signaling pathways through signal transduction mediated by heterodimer formation and autophosphorylation of tyrosine kinases. Moreover, HER2 can form heterodimers with other members of the epidermal growth factor receptor family, playing a significant role in regulating tumor cell proliferation, differentiation, migration, and tumorigenesis. Consequently, it has become a pivotal therapeutic target in the treatment of multiple malignancies, including gastric cancer (2). Globally, HER2-positive gastric cancer patients (immunohistochemical IHC 3+ or IHC 2+ with positive *in situ* hybridization) account for approximately 10% to 20% of all gastric cancer cases (3). The ToGA study demonstrated for the first time that trastuzumab combined with chemotherapy can significantly prolong survival in HER2-positive metastatic gastric cancer patients, ushering in a new era of targeted therapy for gastric cancer (4). However, trastuzumab has significant limitations in its application. On the one hand, it is primarily suitable for HER2-positive populations, while its efficacy is limited in patients with HER2-low expression (IHC 2+/FISH-) (5). On the other hand, although trastuzumab combined with chemotherapy is the standard first-line regimen for HER2-positive gastric cancer, its cross-line efficacy in second-line therapy remains controversial. Some patients with resistance require more effective novel therapeutic agents to improve prognosis.

Antibody-drug conjugates (ADCs) represent a novel generation of targeted therapeutics delivered via macromolecular carriers. These conjugates are formed by covalently linking monoclonal antibodies to cytotoxic drugs through a linker. Their core advantage lies in utilizing the targeting capability of antibodies to precisely deliver cytotoxic drugs to malignant tumor cells, thereby achieving highly specific targeting capability and potent cytotoxic efficacy, which enables accurate elimination of cancer cells (6). Disitamab vedotin (RC48) is the first domestically developed HER2-targeted ADC in China. It is composed of a novel humanized anti-HER2 antibody Disitamab, a protease-cleavable valine-citrulline (VC) linker, and the microtubule inhibitor monomethyl auristatin E (MMAE) (7). After binding to HER2 on the surface of tumor cells, RC48 is efficiently internalized into the cytoplasm. In the acidic lysosomal environment, the VC linker is specifically cleaved by lysosomal proteases to release active MMAE. MMAE blocks tubulin polymerization, arrests tumor cells in the G2/M phase, and induces apoptosis. Moreover, membrane-permeable MMAE can diffuse into adjacent tumor cells and exert a potent bystander killing effect independent of HER2 expression status, which enables RC48 to exert favorable antitumor activity in both HER2-positive and HER2-low-expressing gastric cancer (8). The structure and mechanism of RC48 are illustrated in **Supplementary Figure 1**. Existing studies have shown that when RC48 was used as third-line or later-line therapy for patients with HER2-overexpressing advanced gastric cancer, the objective response rate (ORR) was 24.8%, with a median progression-free survival (mPFS) of 4.1 months and a median overall survival of 7.9 months, demonstrating favorable survival benefits. It has been approved for the treatment of HER2-positive gastric cancer in the third-line setting (9, 10). Although RC48 has shown promising efficacy in patients with advanced gastric cancer, its safety and effectiveness in real-world clinical practice require further validation through real-world evidence. Therefore, this study performed a retrospective analysis to investigate the real-world efficacy and safety of RC48 in patients with HER2-expressing (including patients with IHC 1+, 2+, and 3+) advanced gastric cancer and to assess factors influencing clinical outcomes.

## 2 Materials and methods

### 2.1 Study design and patients

This was a single-center retrospective study reviewing medical records of HER2-expressing advanced gastric cancer patients treated with RC48 at the Affiliated Cancer Hospital of Nanjing Medical University (Jiangsu Cancer Hospital, Jiangsu Institute of Cancer Research) from October 2021 to October 2024. HER2 expression was assessed in pre-RC48 treatment tumor samples by IHC (with *in situ* hybridization [ISH] for IHC 2+ cases) per the 2023 Guidelines for diagnosis and treatment of gastric cancer from the Chinese Society of Clinical Oncology (CSCO) (10). HER2 overexpression was defined as IHC 3+ or IHC 2+/ISH-positive, and low expression as IHC 1+ or IHC 2+/ISH-negative. Inclusion criteria were as follows: 1) age ≥18 years old; 2) pathologically diagnosed with advanced GC (including gastroesophageal junction adenocarcinoma); 3) Receiving RC48 monotherapy or RC48 combined with chemotherapy/targeted therapy/immunotherapy, with this regimen being a third-line or higher antitumor treatment; 4) complete imaging data before and after medication. Exclusion criteria were as follows: 1) combined with other malignancies; 2) patients with severe and uncontrolled organic lesions who are unable to undergo or tolerate antitumor therapy; 3) missing critical information in medical records. The study was approved by the Ethics Committee of the Affiliated Cancer Hospital of Nanjing Medical University & Jiangsu Cancer Hospital & Jiangsu Institute of Cancer Research (NO. KY-2025-163). Because this study was retrospective and observational in design and all patient data were anonymized, the Ethics Committee waived the requirement for written informed consent.

### 2.2 Treatment

All enrolled patients were treated with Disitamab Vedotin for Injection (manufactured by RemeGen Co., Ltd., Yantai; National Drug Approval Number S20210017), administered via intravenous infusion at a dose of 2.5 mg/kg (calculated based on the patient’s actual body weight), with a dosing interval of once every 2 weeks (or 3 weeks/4 weeks).

### 2.3 Follow-up and clinical endpoints

Patients were followed up per routine clinical practice, typically every 2 treatment cycles. The follow-up period in this study commenced on the date of the patient’s first RC48 treatment and concluded on December 31, 2024. Survival outcomes were censored if the patient remained alive, was lost to follow-up, or died from non-disease-related causes at the end of follow-up. Follow-up methods included outpatient review, re-hospitalization, medical record review, and telephone contact.

Tumor response was evaluated retrospectively by the treating physician based on routinely collected imaging data and assessed according to Response Evaluation Criteria in Solid Tumors (RECIST) version 1.1. The endpoints included complete response (CR), partial response (PR), stable disease (SD), and progressive disease (PD) (11). The ORR = (complete response [CR] + partial response [PR])/total cases×100%, and the disease control rate (DCR)=(CR+PR+SD)/total cases×100%. Progression-free survival (PFS) was defined as the time from the start of treatment to the first disease progression or death, whichever occurred first. The severity of all adverse events was performed according to the Common Terminology Criteria for Adverse Events (CTCAE) version 5.0 (12).

### 2.4 Statistical analysis

Descriptive statistics (percentages, means, and medians) were used to describe the baseline characteristics and clinical features of advanced gastric cancer patients treated with RC48. The chi-square test or Fisher’s exact probability method was employed to compare the DCR among different clinicopathological characteristic groups for categorical data. Survival curves were calculated using the Kaplan-Meier method, with Two-group differences compared by the Log-rank test. A *P-*value < 0.05 was considered statistically significant.

## 3 Results

### 3.1 Patient characteristics and follow-up outcomes

This study enrolled a total of 58 patients with advanced gastric cancer who were treated with RC48. The baseline characteristics were as follows: there were 44 males (75.9%) and 14 females (24.1%); the majority of patients were over 50 years of age (detailed age-stratified data are shown in **Table 1**). According to RECIST criteria, 6 (10.3%), 31 (53.5%) and 21 (36.2%) patients achieved PR, SD and PD, respectively. The ORR was 10.3% and the DCR was 63.8%. As of the follow-up endpoint (December 31, 2024), among the 58 enrolled patients, 12 patients (20.7%) remained alive, 7 patients (12.1%) were confirmed dead, and 39 patients (67.2%) were lost to follow-up (survival status could not be confirmed via outpatient review, re-hospitalization, medical record review, and telephone contact). Detailed individual data for the 7 deceased patients are presented in **Supplementary Table 1**.

**Table 1:** Clinical characteristics of patients and their correlation with DCR.

Characteristics	Patients number, N = 58	DCR[*n*(%)]	*P*
Age/years			
18~49	6	3(50.0)	0.657
≥50	52	34(65.4)	
Gender			
male	44	28(63.6)	0.965
female	14	9(64.3)	
BMI			
> 18.5 kg·m^-2^	23	14(60.9)	0.896
18.5~23.9 kg·m^-2^	33	22(66.7)	
> 23.9 kg·m^-2^	2	1(50.0)	
Treatment regimen			
RC48 monotherapy	16	11(68.8)	0.424
ICIs+RC48	14	6(42.9)	
targeted+RC48	17	11(64.7)	
chemotherapy+RC48	5	4(80.0)	
ICIs+chemotherapy/targeted+RC48	6	5(83.3)	
Cycle length			
2-week regimen	33	26(78.8)	0.018
3-week regimen	22	10(45.5)	
4-week regimen	3	1(33.3)	
Underlying diseases			
yes	21	12(57.1)	0.427
no	37	25(67.6)	
HER2 expression status			
HER2 over expression	27	18(66.7)	0.671
HER2 low expression	31	19(61.3)	
Previous use of trastuzumab			
yes	31	22(80.0)	0.223
no	27	15(55.6)	
Line of RC48 therapy			
3rd line	32	24(75.0)	0.049
>3rd line	26	13(50.0)	
Whether ADR occurred			
yes	30	23(76.7)	0.035
no	28	14(50.0)	

RC48, disitamab vedotin; DCR, disease control rate; ICIs, immune checkpoint inhibitors; HER2, human epidermal growth factor receptor 2; ADR, adverse drug reaction.

### 3.2 Correlation between clinical characteristics and DCR in patients

To analyze the correlation between clinical factors and DCR, univariate analysis was performed using the chi-square test or Fisher’s exact probability test for the following 10 clinical factors: age, gender, BMI, treatment regimen, dosing cycle length, underlying diseases, HER2 expression status, prior use of trastuzumab, line of RC48 therapy, and occurrence of ADRs. The results showed that the difference in DCR among different dosing cycle length was statistically significant, with the 2-week regimen demonstrating a significantly higher DCR than the 3-week regimen (78.8% *vs.* 45.5%, *P* = 0.011). The DCR for RC48 as third-line therapy was significantly higher than that for RC48 as later-than-third-line therapy (75.0% *vs*. 50.0%, *P* = 0.049). Patients who experienced ADR had a significantly higher DCR than those who did not (76.7% *vs*. 50.0%, *P* = 0.035). No statistically significant associations were found between DCR and the remaining seven clinical factors (**Table 1**).

### 3.3 Correlation between clinical characteristics and PFS in patients

**Figure 1:**
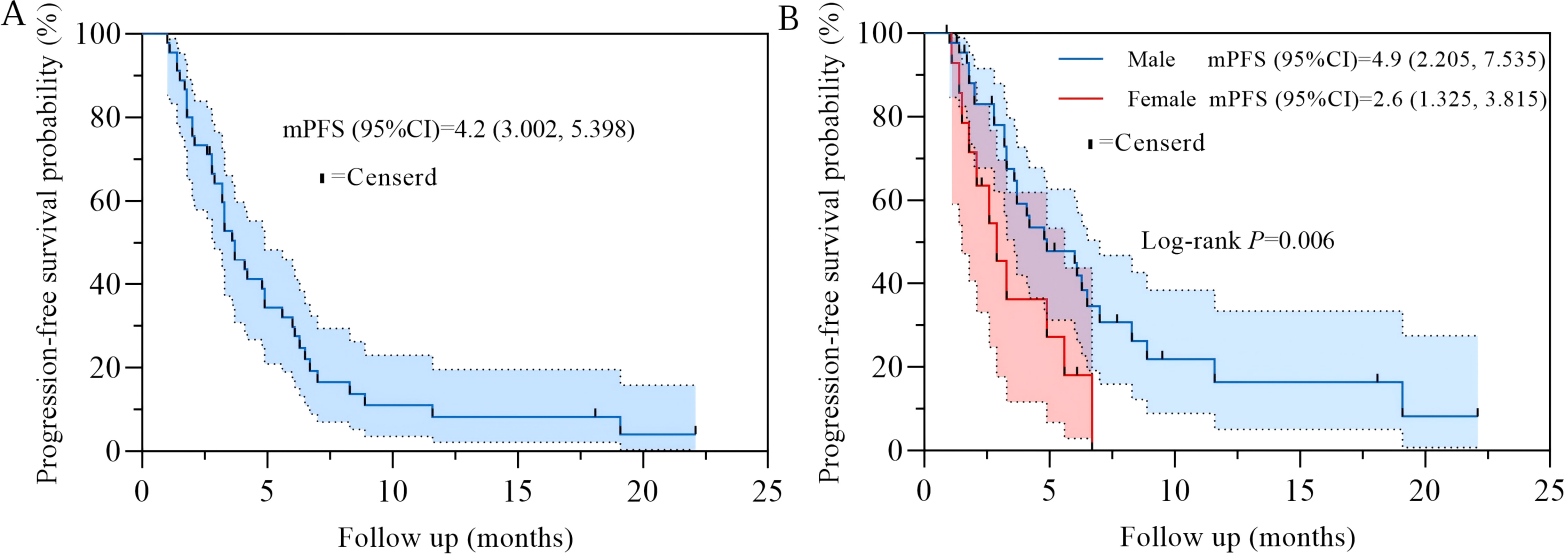
Kaplan-Meier curve of progression-free survival for patients. **(A)** progression-free survival of 58 patients receiving RC48 therapy; **(B)** compare progression-free survival by gender.

**Figure 2:**
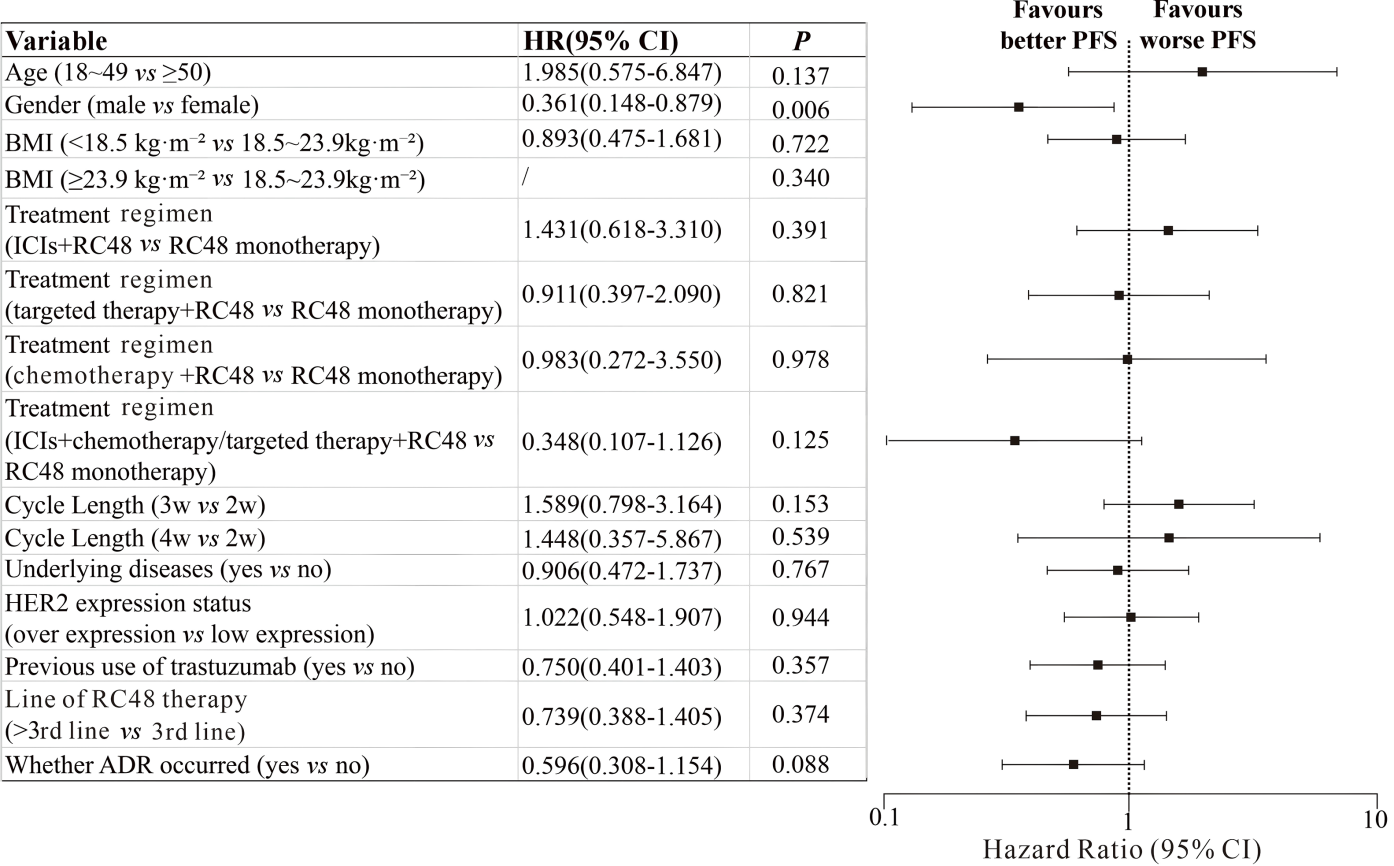
Correlation between clinical characteristics and PFS in patients. HR, hazard ratio; CI, confidence interval; BMI, body mass index; ICIs, immune checkpoint inhibitors; ADR, adverse drug reaction; PFS, progression-free survival; HER2, human epidermal growth factor receptor 2; 2w, 2-week regimen; 3w, 3-week regimen; 4w, 4-week regimen.

Among 58 patients who received line 3 or above RC48 therapy, the mPFS was 4.2 months (95% CI: 3.002~5.398) (**Figure 1A**). Subgroup survival analysis revealed that in terms of gender difference, male patients with advanced gastric cancer treated with RC48 exhibited a significantly longer mPFS than female patients (male *vs* female; HR = 0.471, 95% CI: 0.199-1.118, *P* = 0.006, **Figures 1B**, **2**). Furthermore, univariate analysis of PFS showed that whether ADR occurred (yes *vs* no; HR = 0.596, 95% CI: 0.308-1.154) were potential risk factors for PFS, but there was no statistical significance (*P* = 0.088). Other characteristics, including age, BMI, treatment regimen, cycle length, underlying diseases, HER2 expression status, previous use of trastuzumab, RC48 treatment number of lines, showed no statistically significant association with mPFS, as detailed in **Figure 2**. No significant association between specific types of underlying diseases and PFS was observed in the exploratory subgroup analysis. In addition, during the entire follow-up period, neither RC48 treatment-related acute exacerbation of chronic underlying diseases, nor clinical remission or cure of these non-neoplastic comorbidities was observed.

### 3.4 Safety

The ADRs identified in this study are listed in **Table 2**. RC48-related ADRs occurred in 30 patients (51.7%), with a cumulative total of 67 ADRs of all severity levels. According to the CTCAE version 5.0 grading criteria, there were 10 cases of grade III or higher ADRs. The primary manifestations included decreased hemoglobin (4 cases), neutropenia (3 cases), gastrointestinal toxicity (nausea and vomiting, 1 case), dermal toxicity (herpes zoster, 1 case), and hyperuricemia (1 case) (**Table 2**). During the treatment period, RC48 dose reduction due to ADRs was required in 3 patients (5.2%). For the remaining patients who experienced ADRs, all symptoms were alleviated or improved following symptomatic treatment. No treatment discontinuation occurred due to RC48-related ADRs. Furthermore, no RC48-related severe organ failure or irreversible organ function impairment was observed in this study during the entire follow-up period.

**Table 2:** RC48 treatment-related adverse reactions (*n* = 67).

Adverse events	ADR Grading	
	All grade [n(%)]	≥3 grade [n(%)]
Neutropenia	14(20.9)	3(4.5)
Leucopenia	4(6.0)	0
Thrombocytopenia	9(13.4)	0
Hemoglobin reduction	11(16.4)	4(6.0)
Liver impairment	14(20.9)	0
Cardiovascular toxicity	6(9.0)	0
Digestive tract toxicity	5(7.5)	1(1.5)
Cutaneous toxicity	1(1.5)	1(1.5)
Elevated uric acid^*^	2(3.0)	1(1.5)
Hyperuricemia	1(1.5)	0

^*^ Adverse drug reactions not recorded in the specification.

The 58 patients were divided into an ADR group and a non-ADR group based on whether RC48 treatment-related ADRs occurred. Univariate analysis revealed that the differences between the two groups in the indicators of “presence of underlying diseases” and “line of RC48 therapy” were statistically significant (*P* < 0.05). No statistically significant differences were observed between the groups for the other indicators (*P >*0.05), as shown in **Table 3**.

**Table 3:** Univariate analysis of treatment-related adverse reactions in RC48 [*n* (%)].

Variable		ADR (*n* = 30)	Non-ADR (*n* = 28)	χ^2^/ Fisher	*P*
Age/years	18~49	4(13.3)	2(7.1)	/	0.671
	≥50	26(86.7)	26(92.9)		
Gender	Male	22(73.3)	22(78.6)	0.217	0.641
	Female	8(26.7)	6(21.4)		
BMI	<18.5 kg·m^-2^	13(43.3)	10(35.7)	0.610	0.789
	18.5~23.9 kg·m^-2^	16(53.3)	17(60.7)		
	>23.9 kg·m^-2^	1(3.3)	1(3.6)		
Treatment regimen	RC48 monotherapy	10(33.3)	6(21.4)	1.644	0.846
	ICIs+RC48	6(20.0)	8(28.6)		
	targeted+RC48	9(30.0)	8(28.6)		
	chemotherapy+RC48	2(6.7)	3(10.7)		
	ICIs+chemotherapy/targeted+RC48	3(10.0)	3(10.7)		
Cycle length	2-week regimen	19(63.3)	14(50.0)	1.812	0.445
	3-week Regimen	9(30.0)	13(46.4)		
	4-week Regimen	2(6.7)	1(3.6)		
Underlying diseases	Yes	15(50.0)	6(21.4)	5.119	0.024
	No	15(50.0)	22(78.6)		
HER2 expression status	HER2 over expression	16(53.3)	11(39.3)	1.149	0.284
	HER2 low expression	14(46.7)	17(60.7)		
Previous use of trastuzumab	Yes	17(56.7)	14(50.0)	0.259	0.611
	No	13(43.3)	14(50.0)		
Line of RC48 therapy	3rd line	21(70.0)	11(39.3)	5.524	0.019
	>3rd line	9(30.0)	17(60.7)		

RC48, disitamab vedotin; ICIs, immune checkpoint inhibitors; ADR, drug adverse reactions; HER2, human epidermal growth factor receptor 2.

## 4 Discussion

The combination of 5-fluorouracil and platinum-based chemotherapy serves as the standard treatment regimen for patients with advanced gastric cancer (10). HER2, as a crucial biomarker and key driver gene in gastric cancer, is closely associated with tumor initiation, progression, and metastasis. Currently, the combination of chemotherapy with immune checkpoint inhibitors (ICIs) and HER2-targeted therapy has emerged as a new direction in first-line treatment for advanced gastric cancer, significantly improving patient survival. However, further research is still needed on the therapeutic pathways after drug resistance develops (13, 14). Treatment options for later-line therapy in advanced gastric cancer are limited, and patients are commonly accompanied by declining performance status. Clinical data indicate that the mPFS typically ranges only from 2 to 3 months, and the overall survival is approximately 5 to 6 months (15). Although anti-angiogenic agents such as apatinib and ICIs have demonstrated benefits for some patients, their applicability and efficacy remain limited (16). ADC drugs achieve highly effective antitumor effects through targeted delivery mechanisms. As a domestically developed anti-HER2 ADC in China, RC48 has demonstrated clinical efficacy in patients with HER2-expressing (including IHC 2+ and 1+) urothelial carcinoma, breast cancer, and gastric cancer (17, 18).

This study retrospectively analyzed the clinical efficacy and safety of RC48 treatment in 58 patients with HER2-expressing advanced gastric cancer. Compared with the RC48-C008 trial, the ORR in this study was relatively lower (10.3% *vs*. 24.8%). However, comparable results were observed in terms of the DCR (63.8% *vs.* 42.4%) and mPFS (4.2 months *vs*. 4.1 months), validating the therapeutic value of RC48 in real-world clinical practice (9). This discrepancy in ORR results may stem from the greater heterogeneity of the patient population included in the real-world study, which encompassed more patients with multiple-line therapy and poorer performance status. Furthermore, the HER2 expression criterion in this study was defined as “expressed”, including patients with IHC 1+, 2+, and 3+, whereas the C008 study primarily enrolled patients with HER2 overexpression (IHC 2+ or 3+).

Correlation analysis between clinical factors and DCR revealed that patients receiving the 2-week regimen had a significantly higher DCR compared to those receiving the 3-week regimen (78.8% *vs.* 45.5%, *P* = 0.011). This may be attributed to the fact that dose-dense administration can maintain a higher trough plasma concentrations, providing more sustained effective drug exposure, thereby more effectively inhibiting rapidly proliferating tumor cell subpopulations and thus minimizing tumor repopulation during treatment intervals. Additionally, the DCR was significantly higher for RC48 administered as third-line therapy compared with later-line therapy (75.0% *vs*. 50.0%, *P* = 0.049). This aligns with the treatment patterns of advanced gastric cancer. As the number of treatment lines progresses, patients may experience declining physical condition, increased tumor burden, and accumulation of drug resistance mechanisms, potentially leading to reduced therapeutic efficacy. These findings suggest that earlier application of RC48 (at the third-line stage) may offer patients superior disease control benefits. Furthermore, patients who experienced ADRs achieved a significantly higher DCR than those without ADRs (76.7% *vs*. 50.0%, *P* = 0.035), and also showed a trend toward longer mPFS (6.1 months *vs.* 3.3 months, Log-rank *P* = 0.088). This phenomenon may be associated with drug exposure levels and interindividual metabolic variability: higher plasma drug concentrations could simultaneously enhance antitumor efficacy and increase the risk of ADRs. This indicates that in clinical practice with RC48, proactive prevention and management of ADRs are essential to ensure safety while maintaining full-dose and full-course treatment as much as possible, thereby maximizing therapeutic benefit.

This study also found that male patients treated with RC48 exhibited a significantly longer mPFS than female patients (4.9 months *vs.* 2.9 months, Log-rank *P* = 0.006). The impact of gender differences on antitumor efficacy may involve multiple factors, including tumor epidemiological characteristics, tumor biology, pharmacokinetic variability, and hormonal influences (19). This finding requires further validation in larger sample sizes, and future prospective clinical trials should incorporate gender-stratified analysis to explore the mechanisms underlying the effect of gender differences on the efficacy of RC48.

Regarding safety, 30 (51.7%) experienced RC48-related ADRs, with a total of 67 all-grade ADRs reported. The incidence of grade III or higher ADRs was 17.2% (10/58), primarily including hematological toxicities (hemoglobin reduction in 4 cases, neutropenia in 3 cases) and non-hematological toxicities (digestive tract toxicity in 1 case, cutaneous toxicity in 1 case, and hyperuricemia in 1 case). Compared with the C008 study, the incidence of cutaneous toxicity (such as alopecia, rash, and pruritus) was lower in the present study, which may be attributed to potential underreporting of mild ADRs in retrospective medical records (9). Notably, this study identified one case of hyperuricemia as a new ADR not documented in the approved drug label for RC48, highlighting the significant value of real-world studies in supplementing drug safety information and refining the spectrum of adverse reactions. Furthermore, univariate analysis revealed statistically significant differences between the ADR and non-ADR groups in terms of “presence of underlying diseases” and “line of RC48 therapy” (*P* < 0.05). This suggests that a patient’s baseline health status and treatment stage may influence the safety and tolerability of RC48. On one hand, patients receiving earlier lines of therapy may exhibit better organ function and physical status, demonstrating greater tolerance to drug toxicity. On the other hand, patients with underlying diseases may be more susceptible to treatment-related ADRs due to differences in physiological condition and metabolic characteristics. These findings indicate that in clinical practice, the risk of ADRs should be comprehensively evaluated by considering both the patient’s underlying disease status and treatment lines, thereby facilitating the development of individualized monitoring and management strategies.

## 5 Conclusions

The real-world evidence provided by this study demonstrates that RC48 exhibits certain clinical efficacy and manageable safety in patients with HER2-expressing advanced gastric cancer. Early application of RC48 may achieve superior disease control, while the occurrence of ADRs is associated with the patient’s underlying diseases and treatment lines, and may correlate with enhanced therapeutic response. Male patients may derive more significant PFS benefit from RC48 treatment. These findings provide valuable references for the clinical application of RC48 in advanced gastric cancer. However, this study has limitations: it is a single-center retrospective study with a relatively small sample size, which may introduce selection bias; additionally, incomplete documentation of some ADRs might affect the accuracy of the safety analysis; the high loss-to-follow-up rate and incomplete recording of the specific cause of death in some patients may affect the comprehensive assessment of the overall survival benefit of RC48. Future research directions may focus on the following aspects: conducting multicenter, large-sample prospective studies to further validate the efficacy and safety of RC48 in real-world settings; exploring biomarkers predictive of RC48 efficacy; investigating the mechanisms underlying gender differences in response to RC48; and establishing a risk prediction model for RC48-related ADRs based on patient baseline characteristics to inform individualized treatment strategies.

## Data Availability

The original contributions presented in the study are included in the article/**Supplementary Material**. Further inquiries can be directed to the corresponding author/s.
