# Supplementary material for: Efficacy and safety of disitamab vedotin in treatment of advanced gastric cancer based on real-world
Source: Front Oncol. 2026 May 20;16:1807824. doi: 10.3389/fonc.2026.1807824 (PMC13229803; doi:10.3389/fonc.2026.1807824)
Supplement: Supplementary file 1 [file DataSheet1.pdf]

Supplementary Materials

Manuscript number: 1807824

Title: Efficacy and safety of disitamab vedotin in treatment of advanced gastric cancer based on real-world

Authors: Pan Cheng[1†], Jianjuan Ge[3†], Shuangjia Huang[2], Dongzan Yang[4], Shining Xie[5], Bowen Wang[6], Zhaoshi Bai[2], Jichen He[1*], Xiaolin Liu[2*]

[1]*Department of Pharmacy, Jingjiang People's Hospital Affiliated to Yangzhou University, Taizhou, China*
[2]*Jiangsu Cancer Hospital/The Affiliated Cancer Hospital of Nanjing Medical University/Jiangsu Institute of Cancer Research, Nanjing, China*
[3]*Department of Oncology, Affiliated Tumor Hospital of Nantong University, Nantong, China*
[4]*Department of Pharmacy, Puer People's Hospital, Puer, China*
[5]*Department of Pharmacy, Beijing Ditan Hospital Affiliated to Capital Medical University Xuzhou Hospital (Xuzhou Seventh People's Hospital), Xuzhou, China*
[6]*Department of Pharmacy, Zhoukou First People's Hospital, Zhoukou, China*

Correspondence[*]:
Xiaolin Liu
Email: liu_xl440@163.com
Jichen He
Email: 18256569676@163.com
[†]These authors contributed equally to this work and share first authorship

Journal: Frontiers in Oncology

Supplementary Figure

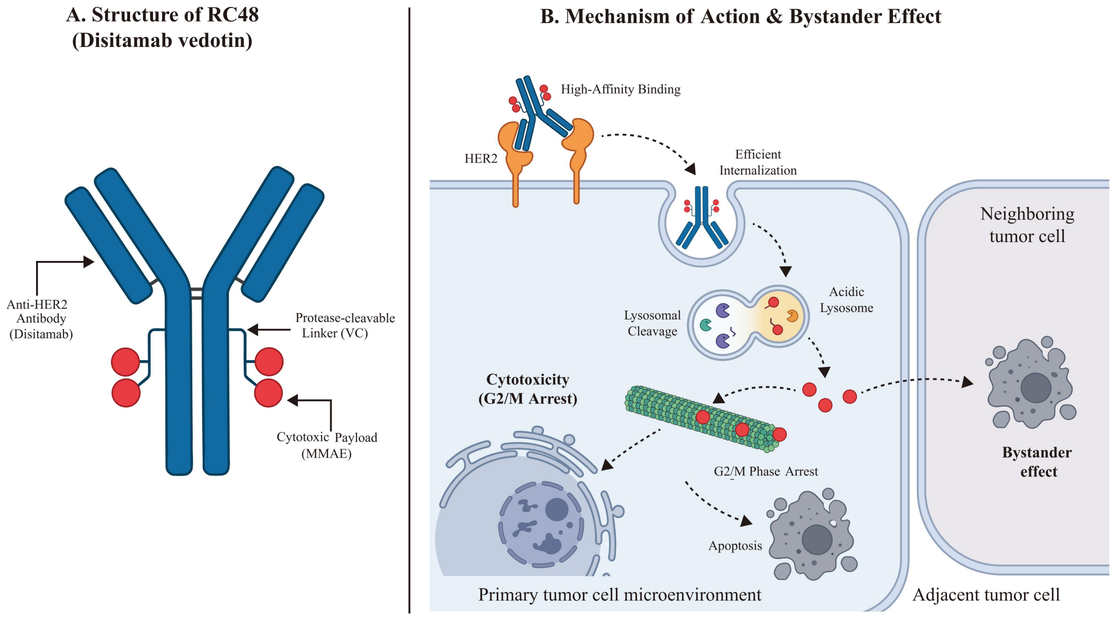

Supplementary Figure S1 Schematic structure and mechanism of action of RC48

A: The core structural components of the anti-HER2 antibody-drug conjugate RC48, including the targeting antibody Disitamab, protease-cleavable VC linker, and cytotoxic payload MMAE; B: The anti-tumor mechanism of RC48, including high-affinity HER2 binding, ADC internalization, lysosomal cleavage and payload release, induction of G2/M phase arrest and apoptosis in target tumor cells, as well as the bystander effect on adjacent tumor cells. HER2: human epidermal growth factor receptor 2; ADC: antibody-drug conjugate; VC: valine-citrulline; MMAE: monomethyl auristatin E.

Supplementary Table

Supplementary Table 1 Detailed information of death events during follow-up

Patient No.	Gender	Age /years	Line of RC48 therapy	PFS /months	Overall survival duration /months	Cause of death	Tumor progression status before death	New non-gastric cancer diseases diagnosed after RC48 treatment
1	Male	70	3rd line	6.3	10.6	Unknow n	PD	Thrombocytop enia
2	Male	43	3rd line	14.3	14.3	Acute cerebral infarctio n	SD	Acute cerebral infarction diagnosed at the time of death, no other new chronic diseases
3	Female	34	3rd line	4.9	16.0	Unknow n	PD	Liver impairment
4	Male	74	3rd line	4.6	5.4	Unknow n	PD	Elevated uric acid
5	Male	57	>3rd line	5.6	5.6	Unknow n	SD	None
6	Female	67	3rd line	2.0	2.0	Unknow n	SD	None
7	Male	68	3rd line	3.3	4.0	Unknow n	PD	Thrombocyto penia

Unknown: data not retrievable from medical records or telephone contact; RC48: disitamab vedotin; PFS: progression-free survival; PD: progressive disease; SD: stable disease

Table note: This table lists the per-patient detailed clinical information of all death events during the follow-up duration, including overall survival duration (months), specific cause of death (disease-related/non-disease-related), tumor progression/recurrence status, and newly diagnosed

diseases other than gastric cancer after RC48 treatment. All data are fully consistent with the survival and safety results in the main manuscript.
